# Medical Society, London, 1802

**Published:** 1802-04

**Authors:** 


### MEDICAL SOCIETY, LONDON, 1802.

ON Monday, March 8, the Medical Society of London, held their Anniversary Meeting, at their house in Bolt Court, Fleet-street, when the Officers and Council for the current year were elected, and an Oration delivered by the President, of which the following Sketch will discover the suitableness and importance.

He began with lamenting the occasion of his unexpected appearance before the Society at that time, the sudden and serious illness of Dr. Garnet, who had been previously appointed to deliver the Anniversary Oration, and to whose ingenuity, industry, and urbanity, he gave just and highly merited commendation.

He then proceeded to the immediate object of his discourse, which was to point out the various studies requisite to form the Medical Practitioner in general, but especially the Physician, whom he had more particularly in view.

He began with preparatory ftudies, and after the knowledge of the mother tongue, he confidered intimate acquaintance with the Latin language as not only defirable, but abfolutely neceffary, both in its Claffical and in its various inferior fpecies of Latinity, in which latter the works of the moft valuable ancient medical authors are written. A moderate knowledge of the Greek language was recommended, but this he ftated to be of inferior importance, there being in general excellent verfions of the moft efteemed authors in that tongue. The French, both as an univerfal language, and as that of fafhion and accomplifhment, muft by no means be neglected, to which may, perhaps, be added the German and Italian.

He then proceeded to the Sciences connected with Medicine, and after general knowledge of the Mathematics, he mentioned fome of the branches of Natural Philofophy, fuch as Mechanics, Hydraulics, Pneumatics, Aftronomy, Electricity, and above all Chemiftry. Natural Hiftory he confidered not only as effentially neceffary to the Student, but as equally interefting and amufing, particularly when not confined to the external appearances of the feveral fubjects of this fcience, but extended to the internal ftructure and ufes of them.

Having gone through thefe preliminary ftudies, he took a view of thofe which more directly relate to the practice of Medicine. He began with Anatomy, a fcience of prime importance, not only to Surgery, but alfo to the cure of internal difeafes; after which Phyfiology was mentioned, then Pathology, which although the moft ufeful branch, is often moft neglected. The extenfive advantages of the ftudy of Materia Medicæ and Pharmacy were fuitably noticed. In his obfervations on the methods of ftudying the Practice of Medicine, he made feveral remarks on the prefent mode of conducting Clinical Lectures, which he confidered as having departed from their original defign of examining and defcanting upon fymptoms by the bed-fide of the patient, to a mere fyftematic hiftory of particular difeafes, and thus proportionably diminifhed in their utility to the Student.

Having next confidered the Practice of Phyfic as conducted by Apothecaries, Accoucheurs and Surgeons, he concluded with feveral very judicious reflections on the religious, moral, and prudential conduct of the Phyfician, an attention to which are unqueftionably greatly conducive to fuccefs and refpectability.

The Names of the Officers and Council were then read over, as alfo were the Prize Queftions for the three enfuing Years, after which the Society adjourned.

### OFFICERS AND COUNCIL OF THE MEDICAL SOCIETY OF LONDON,

For the YEAR 1802.

PRESIDENT.

Dr. James Sims

TREASURER.
Dr. Walker

LIBRARIAN.
Mr. Hurlock

SECRETARIES.
Mr. Field
Mr. J. H. Hooper

SECRETARY FOR FOREIGN CORRESPONDENCE.
Dr. R. Hooper

### COMMITTEES.

I. THEORY and PRACTICE of PHYSIC.
Sir John Macnamara Hayes, Bart.
Dr. Lettſom
Dr. Hulme
Dr. Saunders
Dr. Bradley

II. ANATOMY and PHYSIOLOGY.
Dr. Relph
Dr. Jenner
Mr. Abernethy
Mr. A. Cooper
Mr. Macartney

III. SURGERY.

Mr. Ford

Mr. Norris

Mr. Ware

Mr. Forſter

Mr. Blair

IV. MIDWIFERY.
Dr. John Sims
Dr. Haighton
Mr. Ridout
Mr. Webb
Mr. Ring

V. MATERIA MEDICA and PHARMACY.
Dr. Yelloly
Dr. Marcet
Dr. Curry
Mr. Jackſon
Mr. Jeaffreſon

VI. BOTANY and NATURAL HISTORY.
Dr. Bancroft
Dr. Elliot
Dr. Pearſon
Dr. Rogers
Mr. Chamberlaine

VII. NATURAL PHILOSOPHY and CHEMISTRY
Dr. Babington
Dr. Pinckard
Mr. Parkinſon
Mr. Seaton
Mr. Andree

ANNIVERSARY ORATION for 1803.
Dr. Garnett

REGISTER.
Mr. Spry

### PRIZE MEDALS *to be given by the* MEDICAL SOCIETY *of London.*

THE Society refolve to give annually, a Gold Medal, called the FOTHERGILLIAN MEDAL, of Ten Guineas value, to the Author of the beft Effay on a given Subject; to which the Learned of all Nations are invited as Candidates.

The Medal for the year 1803, will be adjudged to the Author of the beft Differtation on the following fubject: "The "diftinguifhing Characters of CARCINOMATOUS TUMOURS, "and the moft advantageous Mode of treating them."

Subject for the year 1804. "TUSSIS CONVULSIVA, or "HOOPING COUGH, its Hiftory and moft advantageous Treat-"ment, diftinguifhing particularly the Effects of the moft com-"mon and popular Remedies employed for its Cure."

For the year 1805. "CONTAGION; and the Means of "preventing it."

N. B. It is defired that every Answer may, as far as poffible, be founded upon actual Experiments, or well authenticated Facts.

### *Regulations respecting the Medal.*

1. Each Differtation fhall be delivered to the Secretary in the Latin, Englifh, or French Language, on or before the firft day of November, in the preceding year; and the Adjudication of the Medal fhall take place in the laft week of the enfuing February.

2. With each Differtation fhall be delivered a fealed packet with fome motto or device on the outfide; and within, the Author's name and defignation; and the fame motto or device fhall be put upon the Differtation, that the Society may know how to addrefs the fuccefsful Candidate.

3. No Paper with the name of the Author affixed, can be received; and if the Author of any Paper fhall difcover himfelf to the Council, or to any Member thereof, fuch Paper fhall be excluded from all Competition for the Medal.

4. All the Differtations, the fuccefsful one excepted, fhall be returned, if defired, with the fealed packets unopened.

*** The Society propofe to give two Silver Medals annually; one of which fhall be adjudged for the beft Effay, read before the Society within the year, written by a FELLOW; the other for the beft Effay, by a CORRESPONDING MEMBER; or by any perfon NOT a Member of the Society.

